# Supplementary material for: Does political participation help improve the life satisfaction of urban residents: Empirical evidence from China
Source: PLoS One. 2022 Oct 6;17(10):e0273525. doi: 10.1371/journal.pone.0273525 (PMC9536587; doi:10.1371/journal.pone.0273525)
Supplement: Attachment — Submitted filename: Review on the manuscript15.05.2022.pdf [file pone.0273525.s001.pdf]

Review on the manuscript

Title: Does political participation help improve the life satisfaction of urban residents: Empirical evidence from China

Journal: PLOS ONE

10.05.2022

The article is dedicated to the discussion of political participation as a factor to improve life satisfaction in urban China. The article is well structured and gives a clear idea of the research question and the appropriate methodology that is used by the authors. The literature review provides a deep analysis of the existing studies and serves as a good ground for the theoretical explanations of the hypotheses.

The article adds to the literature and methodological discussion since it covers the aspect of urban population life satisfaction testing the political participation of educated and non-educated groups of the population and more.

The manuscript can generate wide public interest and attract many readers. It fits the scope of the journal, and it is ready for publication in the journal after addressing a few concerns mentioned below.

Major concerns

- It is not very clear how the authors address the problem of endogeneity they discuss on page 8 (reversed causality). If there is evidence of that in the literature, how do authors address it in their study, and why do they keep the logic of the causality from political participation to life satisfaction?
- Why political participation is measured as a dummy variable? Why not index? Would such an index that counts the number of different political participation (form 7 items) be of better indicator to reflect political participation?
- Probably it will make sense to estimate the logit or probit model since the dependent variable has an ordered scale (not OLS). Especially for the fact to compare it with PSM logit later. Comparison of OLS with PSM logit would not be that correct if compare ordered logit with PSM logit.
- In the Data description section the authors stated that the data has a total of 10243 individuals, and the urban population is 2577, why not have estimations of the non-urban population to see if political participation in rural areas has any impact on life satisfaction? Later on page 20 the authors are placing that comparison actually, but initially, it is not discussed either in the introduction or in the methodology, not even in the data description.
- The authors have analysis for the employment variable but do not have hypotheses on that. Moreover, it is better to organize the results in the same sequence as the hypotheses were discussed. First on education and then CPC…
- What are the possible effects of digitization of society (including online political participation) on life satisfaction could be?
- It is not clear why we need table 5 if the results of the two last columns are never used in discussions or conclusions. Why not just keep Ologit? Moreover, the final results used for the conclusions are from Ologit model, not from PSM – why? it would be nice to have a clear statement in the conclusions about solving the endogeneity problem and use the results of political participation as the predictor of life satisfaction not Ologit, maybe?
- Overall the paper would gain if the text would be tighter in terms of matching between the sections, especially the results and methodology/hypotheses part.

Minor comments

line 213 – better to use word – respondents/individuals – instead of the word – samples

is it possible to control for social capital (trust, friends, relations, social media engagement) since those variables were proved to be strong life satisfaction determinants?

Table 5 – better to name the models accordingly as OLS, Ologit, PSM not 1,2,3 so that the reader can understand better, or give a not which model is what under the table.

Table 6 – why do you need a gender column? The authors did not have a hypothesis on that.
