# Supplementary material for: Does political participation help improve the life satisfaction of urban residents: Empirical evidence from China
Source: PLoS One. 2022 Oct 6;17(10):e0273525. doi: 10.1371/journal.pone.0273525 (PMC9536587; doi:10.1371/journal.pone.0273525)
Supplement: Attachment — Submitted filename: Response to Reviewers.docx [file pone.0273525.s003.docx]

We deeply appreciate the helpful comments from the two reviewers on the original manuscript. We have carefully considered the comments and have revised the manuscript accordingly. A point-by-point discussion of each comment follows.

**Reviewer1:**

1. The authors did good job by adjusting the text to all the mentioned points. Just one more minor thing is left before publication: proofreading for the grammar mistakes and typo like "Meavy political participation" in Table 8 - means Heavy or involved political participation?

**Response:** We thank the reviewer for this observation. We have revised the manuscript to address grammatical errors, language issues and awkward construction.

2. Also PSM - this is the comparison between urban and nonurban? Correct? Why then the data description is made still only for urban sample? As if the non-urban population is cut from the analysis but then it somehow appeared in the results discussion.

**Response:** We thank the reviewer for this pertinent comment. We apologize for the ambiguity in our original manuscript regarding the role of PSM and the comparison between urban and nonurban. PSM compared the difference in life satisfaction between political and non-political participants in urban areas, rather than comparing urban and rural residents. Our empirical analyses were based on an urban sample, with the exception of further analysis concerning urban-rural comparison. The discussion on non-urban population originated from a reviewer's suggestion that we should add this section to the original manuscript. We followed this suggestion and compared the differences in political participation between urban residents and rural residents in our further analysis. The sample for this part of the comparison temporarily included non-urban populations. Otherwise, our study was restricted to the urban sample.
